# Crystal structure of dipotassium *N*-carbodi­thio­ato-l-prolinate trihydrate

**DOI:** 10.1107/S2056989017011999

**Published:** 2017-08-25

**Authors:** Phil Liebing

**Affiliations:** a ETH Zurich, Laboratory for Inorganic Chemistry, Vladimir-Prelog-Weg 1-5, 10, 8093 Zurich, Switzerland

**Keywords:** crystal structure, proline, amino acid, di­thio­carbamate, π-coordination, coordination polymer, hydrogen bond

## Abstract

The mol­ecular and crystal structure of the l-proline-derived di­thio­carbamate–carboxyl­ate compound poly[tri-μ-aqua-(μ-2-carboxyl­atopyrrolidine-1-carbodi­thio­ato)dipotassium], [K_2_(C_6_H_7_NO_2_S_2_)(H_2_O)_3_]_*n*_ or K_2_(SSC–NC_4_H_7_–COO)·3H_2_O, has been determined. The di­thio­carbamate moiety displays a unique coordination mode, comprising a ‘side-on’ π-coordinated K^+^ cation besides a commonly σ-chelated K^+^ cation. By bridging coordination of the CSS group, COO group and water mol­ecules, the K^+^ cations are linked into a two-dimensional coordination polymer extending parallel to the *ab* plane. These layers are again inter­connected by O—H⋯S hydrogen bonds.

## Abstract 2

K_2_(SSC-NC_4_H_7_—COO)·3H_2_O or C_6_H_13_K_2_NO_5_S_2_ exbibits a highly complex supra­molecular structure in the crystal, which is governed by bridging O– and S-atom coordination, as well as hydrogen bonding.

## Chemical context   

Natural amino acids react readily with carbon di­sulfide in an alkaline environment to give di­thio­carbamate-functionalized carboxyl­ates. Since the first report on a series of barium salts in the 1950s (Zahradnik, 1956 ▸), numerous transition metal complexes have been explored. More recently, various late transition metal complexes of this family have been investigated due to their biological activity (*e.g.* Giovagnini *et al.*, 2005 ▸; Cachapa *et al.*, 2006 ▸; Nagy *et al.*, 2012 ▸). In most cases, the di­thio­carbamate moiety acts as a classical small-bite chelate ligand, while the carboxyl­ate group (often esterified) does not contribute to metal coordination. The structural chemistry of main group derivatives of di­thio­carbamate-derived amino acids is much less explored, even though alkali metal and alkaline earth metal salts are frequently used as precursors for other metal complexes. A key inter­mediate in our ongoing reasearch on coordination polymers with di­thio­carbamate–carboxyl­ates is the l-proline-derived potassium salt K_2_(SSC–NC_4_H_7_–COO). This compound crystallizes from aqueous solution as a trihydrate, which has been structurally characterized in the course of this work.
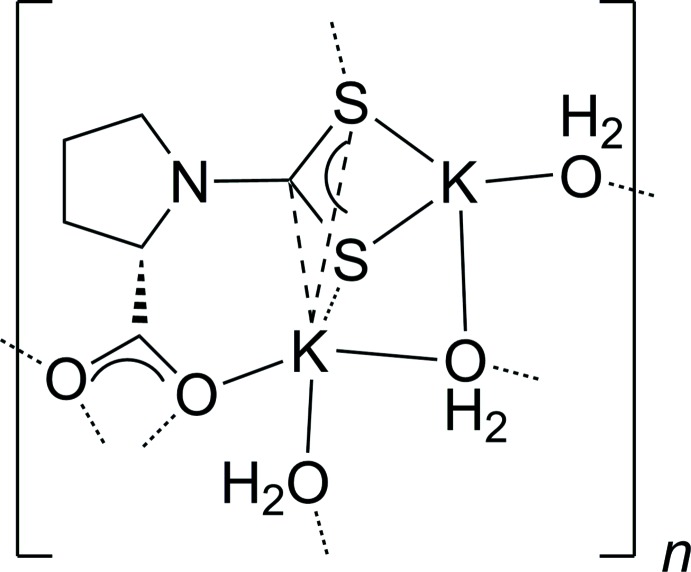


## Structural commentary   

**Figure 1:**
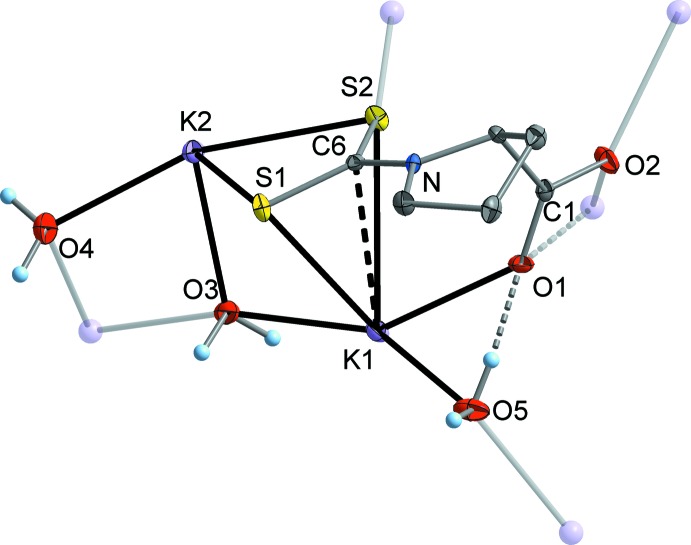
The asymmetric unit of the title compound. Displacement ellipsoids for non-H atoms are drawn at the 50% probability level and H atoms attached to C atoms have been omitted for clarity. Adjacent symmetry-related K^+^ cations are illustrated as semi-transparent spheres.

**Figure 2:**
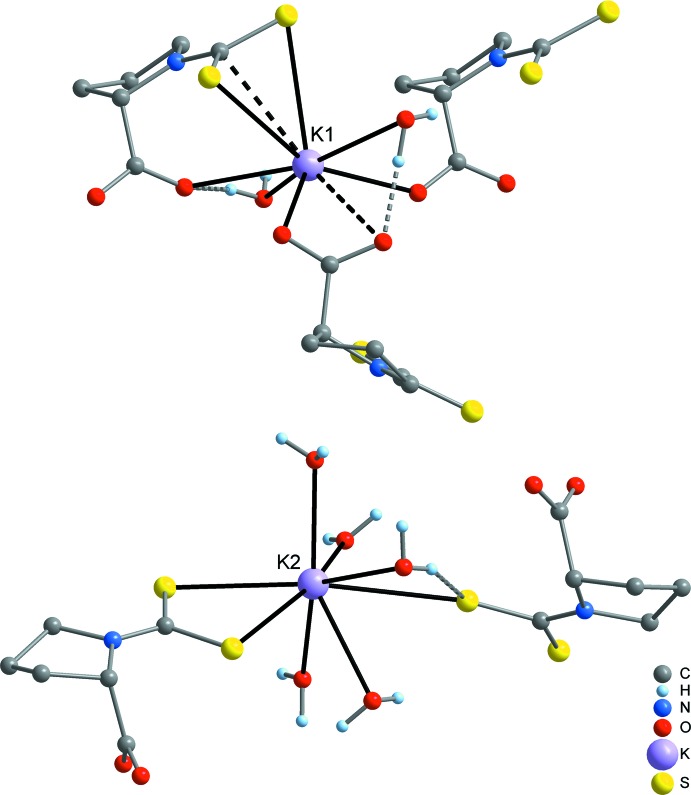
Illustration of the coordination environment of the two K^+^ cations.

**Figure 3:**
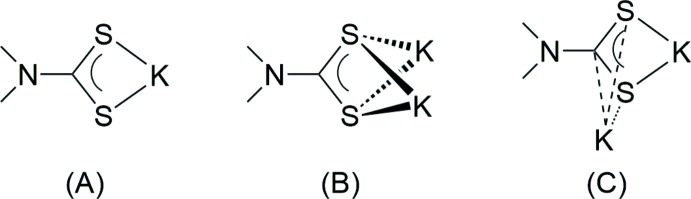
The different coordination modes of the di­thio­carbamate group in potassium complexes: single-chelating (A), symmetric double-chelating (B) and single-chelating combined with π-coordination (this work; C).

The title compound, K_2_(SSC–NC_4_H_7_–COO)·3H_2_O, crystallized as colourless plates in the ortho­rhom­bic space group *P*2_1_2_1_2_1_, with one formula moiety in the asymmetric unit (Fig. 1 ▸). One K atom (K2) is bonded in a typical chelating fashion by the CSS group, while K1 is coordinated ‘side-on’ to the CSS group, certainly under participation of the delocalized π electrons. This rather uncommon coordination mode might be supported by additional coordination of a carboxyl­ate O atom (O1) to K1. K1 adopts a low-symmetric seven-coordination by four carboxyl­ate O atoms, two H_2_O mol­ecules and the π-coordinating CSS group. K2 is eight-coordinated by three S atoms and five H_2_O mol­ecules (Fig. 2 ▸). Consequently, the full coordination mode of the carboxyl­ate group is μ_3_-κ^4^ *O*,*O*′:*O*:*O*′, and the di­thio­carbamate group adopts a μ_3_-κ^6^ *S*,*S*′,*C*:*S*,*S*′:*S* coordination. One H_2_O mol­ecule displays a μ_3_-coordination (O3) and the remaining two H_2_O mol­ecules are coordinating in a μ-bridging mode (O4 and O5). The K—S distances at the σ-chelated K^+^ cation (K2) are 3.2176 (8) and 3.2650 (9) Å, while the K—S separations at the π-coordinated K^+^ cation (K1) are significantly longer at 3.2956 (9) and 3.4463 (8) Å. The coordination mode of the di­thio­carbamate group in the title compound [see (C) in Fig. 3 ▸] is unique, to our knowledge. The most frequently observed coordination pattern in di­thio­carbamates of the heavier alkali metals (K, Rb and Cs) is a symmetric double-chelating mode, leading to a puckered S_2_ *M*_2_ ring (*e.g.* Howie *et al.*, 2008 ▸; Reyes-Martínez *et al.*, 2009 ▸; Mafud, 2012 ▸; see (B) in Fig. 3 ▸]. Nonetheless, the values of the K—S separations in the title compound cover the same range as observed in the reference compounds. A simple chelating coordination with significantly shorter K—S contacts is realized when the K^+^ cation is coordinatively highly saturated, as has been observed in a crown ether complex [Arman *et al.*, 2013 ▸; see (A) in Fig. 3 ▸]. In the title compound, three of the four K—O(carboxyl­ate) contacts are in a range 2.676 (2)–2.802 (2) Å, which is consistent with the values observed in other potassium carboxyl­ates (*e.g.* Ilczyszyn *et al.*, 2009 ▸; Liebing *et al.*, 2016 ▸). However, one contact (K1′—O1) is strongly elongated to 3.358 (2) Å. The K—O(H_2_O) bond lengths cover a range of 2.723 (2)–3.065 (3) Å.

## Supra­molecular features   

**Figure 4:**
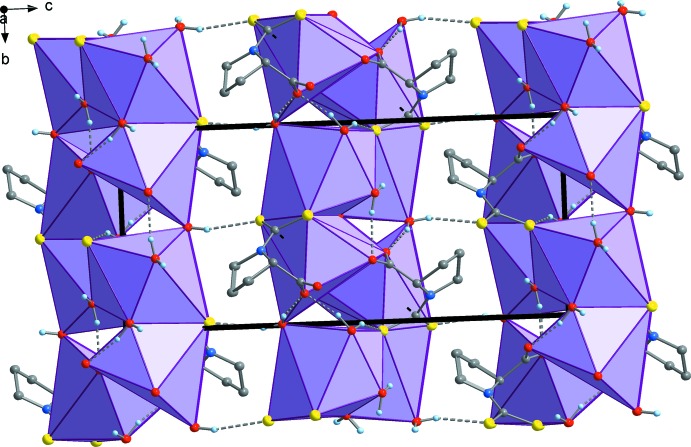
Supramolecular crystal structure comprising polymeric layers extending parallel to (001), viewed in a projection on (100). The bold black lines mark the unit-cell dimensions.

As a result of the bridging coordination of the carboxyl­ate group, the di­thio­carbamate group and the water mol­ecules, a two-dimensional polymeric structure parallel to the *ab* plane is built (Figs. 4 ▸ and 5 ▸). This arrangement is likely supported by O3—H⋯O1^i^, O3—H⋯S2^ii^, O4—H⋯O2^iii^ and O5—H⋯O1 hydrogen bonds within the layer (Table 1 ▸). The layer surfaces are defined by the hydro­phobic hydro­carbon backbones, but additionally the two-dimensional arrays are apparently inter­connected by O5—H⋯S1^iv^ hydrogen bonds.

## Database survey   

For other potassium di­thio­carbamates, see *e.g.* Cambridge Structural Database (CSD; Groom *et al.*, 2016 ▸) refcode AGEHIF (Arman *et al.*, 2013 ▸), KOLLIH (Howie *et al.*, 2008 ▸), LEHRUN (Mafud, 2012 ▸).

For other potassium carboxyl­ates, see *e.g.* CONWOS (Ilczyszyn *et al.*, 2009 ▸), and BIFMIN01 and BIFMUZ01 (Liebing *et al.*, 2016 ▸).

## Synthesis and crystallization   

A slight excess of carbon di­sulfide (approximately 4 ml, 0.06 mol) was added to a solution of l-proline (5.76 g, 0.05 mol) and potassium hydroxide (5.61 g, 0.10 mol) in 30 ml water and the resulting solution was stirred vigorously overnight. The yellow solution obtained was filtered and reduced to dryness *in vacuo*. The crystalline residue was washed with several portions of tetra­hydro­furan and diethyl ether, and dried *in vacuo*, providing analytically pure K_2_(SSC–NC_4_H_7_–COO)·3H_2_O in almost qu­anti­tative (>95%) yield as colourless to light-brown low-melting plates, which are very soluble in water. Single crystals suitable for X-ray structure analysis were obtained by slow evaporation of a concentrated aqueous solution at room temperature. IR: 3372 (*s br*), 3226 (*sh br*), 2985 (*m*), 2949 (*m*), 2875 (*w*), 1641 (*sh*), 1603 (*sh*), 1587 (*s*), 1497 (*s*), 1443 (*s*), 1374 (*s*), 1338 (*m*), 1316 (*w*), 1290 (*s*), 1257 (*m*), 1230 (*w*), 1176 (*m*), 1155 (*s*), 1083 (*w*), 1050 (*w*), 1003 (*m*), 948 (*m*), 918 (*m*), 899 (*w*), 846 (*m*), 794 (*m*), 666 (*s br*), 562 (*s*), 479 (*s*), 446 (*m*) cm^−1. 1^H NMR [400 MHz, D_2_O, 298 (2) K]: δ 1.89–1.98 (3 × *m*, 3H; 3-C*H*_2_ + 4-C*H*_2_), 2.24 (*m*, 1H; 3-C*H*_2_), 3.75 (*m*, 1H; 5-C*H*_2_), 3.83 (*m*, 1H; 5-C*H*_2_), 4.72 (*dd*, *J*_1_ = 8.7, *J*_2_ = 3.2 Hz, 1H; 2-C*H*). ^13^C NMR [100 MHz, D_2_O, 298 (2) K]: δ 24.6 (4-*C*H_2_), 31.5 (3-*C*H_2_), 55.7 (5-*C*H_2_), 69.5 (2-*C*H), 179.9 (*C*OO), 205.8 (*C*SS).

## Refinement   

Crystal data, data collection and structure refinement details are summarized in Table 2 ▸. H atoms on C atoms were fixed geometrically and refined using a riding model, with *U*_iso_(H) = 1.2*U*_eq_(C). C—H distances within the CH_2_ groups were constrained to 0.99 Å and that within the CH group to 1.00 Å. The water H-atom sites were located in difference Fourier maps and refined using restraints on the O—H distance [target value = 0.84 (2) Å]. The corresponding *U*_iso_(H) values were set at 1.5*U*_eq_(O). The reflection (002) disagreed strongly with the structural model and was therefore omitted from the refinement.

## Supplementary Material

Crystal structure: contains datablock(s) I. DOI: 10.1107/S2056989017011999/zl2712sup1.cif

Structure factors: contains datablock(s) I. DOI: 10.1107/S2056989017011999/zl2712Isup2.hkl

CCDC reference: 1569563

Additional supporting information:  crystallographic information; 3D view; checkCIF report

## Figures and Tables

**Figure 5:**
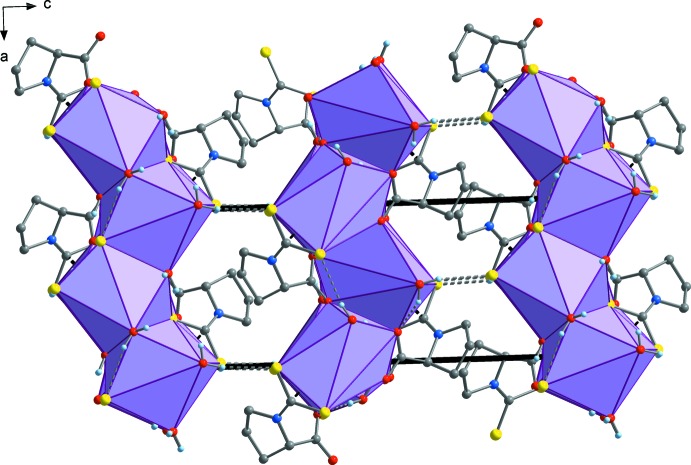
The supra­molecular layer illustrated in Fig. 4 ▸, viewed in a projection on (010).

**Table 1:** Hydrogen-bond geometry (Å, °)

*D*—H⋯*A*	*D*—H	H⋯*A*	*D*⋯*A*	*D*—H⋯*A*
O3—H9⋯O1^i^	0.83 (2)	1.93 (2)	2.752 (3)	170 (4)
O3—H8⋯S2^ii^	0.81 (2)	2.57 (2)	3.3123 (19)	153 (3)
O4—H11⋯O2^iii^	0.80 (2)	2.22 (2)	3.000 (3)	166 (4)
O5—H13⋯O1	0.81 (2)	1.99 (3)	2.724 (3)	150 (3)
O5—H12⋯S1^iv^	0.81 (2)	2.55 (2)	3.341 (2)	163 (3)

Symmetry codes: (i) 
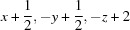
; (ii) 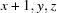; (iii) 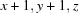; (iv) 
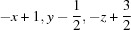
.

**Table 2:** Experimental details

Crystal data	
Chemical formula	[K_2_(C_6_H_7_NO_2_S_2_)(H_2_O)_3_]
*M*_r_	321.49
Crystal system, space group	Orthorhombic, *P*2_1_2_1_2_1_
Temperature (K)	153
*a*, *b*, *c* (Å)	7.1700 (3), 8.9723 (4), 19.8379 (7)
*V* (Å^3^)	1276.20 (9)
*Z*	4
Radiation type	Mo *K*α
μ (mm^−1^)	1.07
Crystal size (mm)	0.44 × 0.10 × 0.07

Data collection	
Diffractometer	Stoe IPDS 2T
Absorption correction	Numerical (*X-AREA* and *X-RED*; Stoe & Cie, 2002 ▸)
*T*_min_, *T*_max_	0.741, 0.935
No. of measured, independent and observed [*I* > 2σ(*I*)] reflections	8831, 2794, 2511
*R*_int_	0.038
(sin θ/λ)_max_ (Å^−1^)	0.639

Refinement	
*R*[*F* ^2^ > 2σ(*F* ^2^)], *wR*(*F* ^2^), *S*	0.023, 0.042, 0.97
No. of reflections	2794
No. of parameters	164
No. of restraints	6
H-atom treatment	H atoms treated by a mixture of independent and constrained refinement
Δρ_max_, Δρ_min_ (e Å^−3^)	0.25, −0.22
Absolute structure	Flack *x* determined using 979 quotients [(*I* ^+^) − (*I* ^−^)]/[(*I* ^+^) + (*I* ^−^)] (Parsons *et al.*, 2013 ▸)
Absolute structure parameter	0.00 (4)

Computer programs: *X-AREA* (Stoe & Cie, 2002 ▸), *X-RED* (Stoe & Cie, 2002 ▸), *SIR97* (Altomare *et al.*, 1999 ▸), *SHELXL2016* (Sheldrick, 2015 ▸), *DIAMOND* (Brandenburg, 1999 ▸) and *publCIF* (Westrip, 2010 ▸).

## supplementary crystallographic information

### Crystal data

**Table:**

[K_2_(C_6_H_7_NO_2_S_2_)(H_2_O)_3_]	*D*_x_ = 1.673 Mg m^−^^3^
*M**_r_* = 321.49	Mo *K*α radiation, λ = 0.71073 Å
Orthorhombic, *P*2_1_2_1_2_1_	Cell parameters from 10736 reflections
*a* = 7.1700 (3) Å	θ = 2.5–29.2°
*b* = 8.9723 (4) Å	µ = 1.07 mm^−^^1^
*c* = 19.8379 (7) Å	*T* = 153 K
*V* = 1276.20 (9) Å^3^	Plate, colorless
*Z* = 4	0.44 × 0.10 × 0.07 mm
*F*(000) = 664	

### Data collection

**Table:**

Stoe IPDS 2T diffractometer	2794 independent reflections
Radiation source: fine-focus sealed tube	2511 reflections with *I* > 2σ(*I*)
Detector resolution: 6.67 pixels mm^-1^	*R*_int_ = 0.038
ω scan	θ_max_ = 27.0°, θ_min_ = 2.5°
Absorption correction: numerical (X-AREA and X-RED; Stoe & Cie, 2002)	*h* = −9→9
*T*_min_ = 0.741, *T*_max_ = 0.935	*k* = −11→11
8831 measured reflections	*l* = −22→25

### Refinement

**Table:**

Refinement on *F*^2^	Hydrogen site location: mixed
Least-squares matrix: full	H atoms treated by a mixture of independent and constrained refinement
*R*[*F*^2^ > 2σ(*F*^2^)] = 0.023	*w* = 1/[σ^2^(*F*_o_^2^) + (0.0175*P*)^2^] where *P* = (*F*_o_^2^ + 2*F*_c_^2^)/3
*wR*(*F*^2^) = 0.042	(Δ/σ)_max_ = 0.001
*S* = 0.97	Δρ_max_ = 0.25 e Å^−^^3^
2794 reflections	Δρ_min_ = −0.22 e Å^−^^3^
164 parameters	Extinction correction: SHELXL2016 (Sheldrick, 2015), Fc^*^=kFc[1+0.001xFc^2^λ^3^/sin(2θ)]^-1/4^
6 restraints	Extinction coefficient: 0.0051 (7)
Primary atom site location: heavy-atom method	Absolute structure: Flack *x* determined using 979 quotients [(I+)-(I-)]/[(I+)+(I-)] (Parsons *et al.*, 2013)
Secondary atom site location: difference Fourier map	Absolute structure parameter: 0.00 (4)

### Special details

**Table:**

Geometry. All esds (except the esd in the dihedral angle between two l.s. planes) are estimated using the full covariance matrix. The cell esds are taken into account individually in the estimation of esds in distances, angles and torsion angles; correlations between esds in cell parameters are only used when they are defined by crystal symmetry. An approximate (isotropic) treatment of cell esds is used for estimating esds involving l.s. planes.

### Fractional atomic coordinates and isotropic or equivalent isotropic displacement parameters (Å^2^)

**Table:**

	*x*	*y*	*z*	*U*_iso_*/*U*_eq_	
C1	0.0231 (3)	0.2133 (3)	0.89993 (13)	0.0120 (5)	
C2	−0.0116 (3)	0.3153 (3)	0.83918 (13)	0.0103 (5)	
H1	−0.098814	0.397715	0.851816	0.012*	
C3	0.2180 (4)	0.3035 (3)	0.74906 (14)	0.0140 (6)	
H3	0.348536	0.268134	0.751636	0.017*	
H2	0.205712	0.372618	0.710395	0.017*	
C4	0.0837 (4)	0.1734 (3)	0.74238 (15)	0.0157 (6)	
H5	0.135307	0.082539	0.763649	0.019*	
H4	0.056006	0.152175	0.694439	0.019*	
C5	−0.0907 (4)	0.2267 (3)	0.77943 (14)	0.0156 (6)	
H6	−0.166526	0.141324	0.795180	0.019*	
H7	−0.168314	0.290872	0.750111	0.019*	
C6	0.2657 (3)	0.4759 (3)	0.84517 (12)	0.0098 (5)	
N	0.1619 (3)	0.3766 (2)	0.81226 (11)	0.0092 (4)	
O1	0.1821 (2)	0.1556 (2)	0.90730 (10)	0.0157 (4)	
O2	−0.1122 (2)	0.1894 (2)	0.93751 (12)	0.0207 (4)	
O3	0.7663 (3)	0.5383 (3)	0.98964 (10)	0.0217 (4)	
H8	0.856 (4)	0.512 (4)	0.9680 (15)	0.033*	
H9	0.747 (4)	0.471 (4)	1.0179 (15)	0.033*	
O4	0.9025 (3)	0.8561 (2)	0.92654 (13)	0.0280 (5)	
H10	0.999 (4)	0.821 (4)	0.912 (2)	0.042*	
H11	0.914 (5)	0.943 (3)	0.934 (2)	0.042*	
O5	0.4779 (3)	0.0059 (2)	0.85399 (10)	0.0240 (5)	
H13	0.373 (3)	0.038 (4)	0.8575 (18)	0.036*	
H12	0.505 (4)	−0.005 (4)	0.8144 (12)	0.036*	
K1	0.53230 (8)	0.28003 (6)	0.93500 (3)	0.01712 (14)	
K2	0.54335 (7)	0.76029 (6)	0.93744 (3)	0.01595 (13)	
S1	0.47870 (8)	0.52667 (8)	0.81344 (3)	0.01487 (14)	
S2	0.18705 (8)	0.54540 (7)	0.92079 (3)	0.01487 (15)	

### Atomic displacement parameters (Å^2^)

**Table:**

	*U*^11^	*U*^22^	*U*^33^	*U*^12^	*U*^13^	*U*^23^
C1	0.0121 (11)	0.0107 (12)	0.0133 (13)	−0.0024 (9)	−0.0008 (10)	−0.0016 (9)
C2	0.0068 (11)	0.0108 (11)	0.0134 (13)	0.0003 (9)	−0.0006 (10)	0.0007 (9)
C3	0.0154 (14)	0.0153 (13)	0.0112 (14)	−0.0017 (10)	0.0030 (10)	−0.0030 (11)
C4	0.0192 (14)	0.0133 (13)	0.0145 (15)	−0.0047 (10)	−0.0023 (10)	−0.0031 (11)
C5	0.0151 (12)	0.0151 (14)	0.0167 (15)	−0.0028 (10)	−0.0038 (10)	−0.0007 (11)
C6	0.0097 (10)	0.0096 (11)	0.0100 (12)	0.0016 (10)	−0.0005 (8)	0.0021 (11)
N	0.0095 (9)	0.0086 (10)	0.0096 (11)	−0.0024 (8)	−0.0003 (8)	−0.0004 (8)
O1	0.0135 (8)	0.0164 (9)	0.0171 (11)	0.0057 (8)	0.0000 (8)	0.0034 (8)
O2	0.0127 (8)	0.0312 (12)	0.0183 (11)	−0.0002 (7)	0.0040 (9)	0.0082 (10)
O3	0.0206 (9)	0.0208 (10)	0.0237 (11)	0.0048 (8)	0.0084 (8)	0.0098 (10)
O4	0.0223 (10)	0.0214 (11)	0.0403 (16)	−0.0048 (8)	0.0009 (10)	−0.0010 (12)
O5	0.0233 (9)	0.0330 (13)	0.0158 (10)	0.0126 (9)	0.0033 (8)	0.0009 (9)
K1	0.0115 (2)	0.0201 (3)	0.0197 (3)	0.0003 (2)	−0.0003 (3)	0.0014 (2)
K2	0.0146 (3)	0.0140 (3)	0.0192 (3)	−0.0005 (2)	−0.0023 (3)	−0.0006 (2)
S1	0.0125 (3)	0.0187 (3)	0.0134 (3)	−0.0058 (3)	0.0023 (2)	−0.0005 (3)
S2	0.0135 (3)	0.0182 (3)	0.0129 (3)	−0.0022 (3)	0.0020 (2)	−0.0062 (3)

### Geometric parameters (Å, º)

**Table:**

C1—O2	1.242 (3)	O3—K1	3.060 (2)
C1—O1	1.261 (3)	O3—H8	0.81 (2)
C1—C2	1.533 (4)	O3—H9	0.83 (2)
C1—K1^i^	3.276 (3)	O4—K2	2.723 (2)
C2—N	1.462 (3)	O4—K2^iii^	3.065 (3)
C2—C5	1.536 (4)	O4—H10	0.81 (2)
C2—H1	1.0000	O4—H11	0.80 (2)
C3—N	1.471 (3)	O5—K2^iv^	2.796 (2)
C3—C4	1.519 (4)	O5—K1	2.964 (2)
C3—H3	0.9900	O5—H13	0.81 (2)
C3—H2	0.9900	O5—H12	0.81 (2)
C4—C5	1.527 (4)	K1—O2^v^	2.6758 (19)
C4—H5	0.9900	K1—O2^vi^	2.747 (2)
C4—H4	0.9900	K1—C1^vi^	3.276 (3)
C5—H6	0.9900	K1—S1	3.2956 (9)
C5—H7	0.9900	K1—O1^vi^	3.358 (2)
C6—N	1.332 (3)	K1—S2	3.4463 (8)
C6—S1	1.714 (2)	K1—H9	2.83 (4)
C6—S2	1.719 (3)	K1—H13	2.90 (4)
C6—K1	3.150 (2)	K2—O5^vii^	2.796 (2)
O1—K1	2.8023 (19)	K2—O3^viii^	3.050 (2)
O1—K1^i^	3.358 (2)	K2—O4^viii^	3.065 (3)
O2—K1^ii^	2.6758 (19)	K2—S2	3.2176 (8)
O2—K1^i^	2.747 (2)	K2—S1	3.2650 (9)
O3—K2	2.756 (2)	K2—S2^iii^	3.4656 (9)
O3—K2^iii^	3.050 (2)	S2—K2^viii^	3.4656 (9)
			
O2—C1—O1	124.5 (2)	C6—K1—K2	57.29 (5)
O2—C1—C2	116.6 (2)	C1^vi^—K1—K2	88.33 (4)
O1—C1—C2	118.9 (2)	S1—K1—K2	48.625 (16)
O2—C1—K1^i^	54.47 (14)	O1^vi^—K1—K2	79.14 (4)
O1—C1—K1^i^	82.72 (15)	S2—K1—K2	47.420 (14)
C2—C1—K1^i^	141.07 (16)	O2^v^—K1—K1^vi^	35.39 (5)
N—C2—C1	111.93 (19)	O2^vi^—K1—K1^vi^	77.43 (4)
N—C2—C5	103.1 (2)	O1—K1—K1^vi^	141.94 (4)
C1—C2—C5	110.9 (2)	O5—K1—K1^vi^	108.66 (4)
N—C2—H1	110.2	O3—K1—K1^vi^	56.26 (4)
C1—C2—H1	110.2	C6—K1—K1^vi^	152.21 (5)
C5—C2—H1	110.2	C1^vi^—K1—K1^vi^	55.88 (5)
N—C3—C4	104.1 (2)	S1—K1—K1^vi^	126.83 (2)
N—C3—H3	110.9	O1^vi^—K1—K1^vi^	39.01 (3)
C4—C3—H3	110.9	S2—K1—K1^vi^	135.23 (3)
N—C3—H2	110.9	K2—K1—K1^vi^	95.725 (18)
C4—C3—H2	110.9	O2^v^—K1—H9	69.9 (7)
H3—C3—H2	109.0	O2^vi^—K1—H9	67.0 (6)
C3—C4—C5	103.7 (2)	O1—K1—H9	147.4 (6)
C3—C4—H5	111.0	O5—K1—H9	152.8 (7)
C5—C4—H5	111.0	O3—K1—H9	15.6 (5)
C3—C4—H4	111.0	C6—K1—H9	108.6 (6)
C5—C4—H4	111.0	C1^vi^—K1—H9	54.5 (5)
H5—C4—H4	109.0	S1—K1—H9	94.6 (5)
C4—C5—C2	103.4 (2)	O1^vi^—K1—H9	35.0 (5)
C4—C5—H6	111.1	S2—K1—H9	91.1 (6)
C2—C5—H6	111.1	K2—K1—H9	51.5 (6)
C4—C5—H7	111.1	K1^vi^—K1—H9	45.5 (6)
C2—C5—H7	111.1	O2^v^—K1—H13	99.1 (5)
H6—C5—H7	109.0	O2^vi^—K1—H13	114.5 (7)
N—C6—S1	119.70 (18)	O1—K1—H13	40.8 (5)
N—C6—S2	119.16 (17)	O5—K1—H13	15.8 (4)
S1—C6—S2	121.09 (15)	O3—K1—H13	166.5 (6)
N—C6—K1	104.05 (16)	C6—K1—H13	83.0 (6)
S1—C6—K1	79.34 (8)	C1^vi^—K1—H13	122.4 (7)
S2—C6—K1	84.71 (9)	S1—K1—H13	94.0 (7)
C6—N—C2	123.2 (2)	O1^vi^—K1—H13	138.6 (7)
C6—N—C3	124.3 (2)	S2—K1—H13	101.0 (5)
C2—N—C3	112.12 (18)	K2—K1—H13	139.7 (7)
C1—O1—K1	132.01 (16)	K1^vi^—K1—H13	122.3 (6)
C1—O1—K1^i^	75.41 (15)	H9—K1—H13	167.8 (8)
K1—O1—K1^i^	92.03 (5)	O4—K2—O3	73.12 (7)
C1—O2—K1^ii^	132.87 (17)	O4—K2—O5^vii^	82.11 (7)
C1—O2—K1^i^	103.94 (16)	O3—K2—O5^vii^	152.81 (6)
K1^ii^—O2—K1^i^	110.25 (8)	O4—K2—O3^viii^	117.80 (6)
K2—O3—K2^iii^	97.35 (7)	O3—K2—O3^viii^	128.88 (4)
K2—O3—K1	95.52 (6)	O5^vii^—K2—O3^viii^	72.82 (6)
K2^iii^—O3—K1	166.87 (8)	O4—K2—O4^viii^	119.29 (6)
K2—O3—H8	118 (2)	O3—K2—O4^viii^	67.29 (6)
K2^iii^—O3—H8	85 (3)	O5^vii^—K2—O4^viii^	137.28 (6)
K1—O3—H8	92 (3)	O3^viii^—K2—O4^viii^	64.51 (6)
K2—O3—H9	133 (2)	O4—K2—S2	158.71 (6)
K2^iii^—O3—H9	103 (2)	O3—K2—S2	93.79 (5)
K1—O3—H9	66 (2)	O5^vii^—K2—S2	106.15 (5)
H8—O3—H9	106 (3)	O3^viii^—K2—S2	83.48 (4)
K2—O4—K2^iii^	97.70 (8)	O4^viii^—K2—S2	67.94 (5)
K2—O4—H10	135 (3)	O4—K2—S1	106.09 (6)
K2^iii^—O4—H10	84 (3)	O3—K2—S1	84.35 (5)
K2—O4—H11	113 (2)	O5^vii^—K2—S1	92.06 (5)
K2^iii^—O4—H11	98 (3)	O3^viii^—K2—S1	130.18 (4)
H10—O4—H11	111 (4)	O4^viii^—K2—S1	113.44 (5)
K2^iv^—O5—K1	108.11 (6)	S2—K2—S1	54.915 (17)
K2^iv^—O5—H13	113 (3)	O4—K2—S2^iii^	67.95 (6)
K1—O5—H13	77 (3)	O3—K2—S2^iii^	83.46 (5)
K2^iv^—O5—H12	116 (3)	O5^vii^—K2—S2^iii^	97.70 (5)
K1—O5—H12	126 (3)	O3^viii^—K2—S2^iii^	60.71 (4)
H13—O5—H12	110 (3)	O4^viii^—K2—S2^iii^	63.57 (5)
O2^v^—K1—O2^vi^	111.87 (5)	S2—K2—S2^iii^	128.38 (2)
O2^v^—K1—O1	137.40 (6)	S1—K2—S2^iii^	167.58 (2)
O2^vi^—K1—O1	83.24 (6)	O4—K2—K1	109.40 (5)
O2^v^—K1—O5	83.31 (6)	O3—K2—K1	44.96 (5)
O2^vi^—K1—O5	122.17 (6)	O5^vii^—K2—K1	141.10 (5)
O1—K1—O5	56.29 (5)	O3^viii^—K2—K1	125.84 (4)
O2^v^—K1—O3	72.61 (6)	O4^viii^—K2—K1	70.31 (4)
O2^vi^—K1—O3	78.76 (6)	S2—K2—K1	52.061 (16)
O1—K1—O3	149.61 (6)	S1—K2—K1	49.238 (17)
O5—K1—O3	153.24 (6)	S2^iii^—K2—K1	121.17 (2)
O2^v^—K1—C6	139.31 (7)	O4—K2—K2^iii^	44.10 (5)
O2^vi^—K1—C6	103.67 (6)	O3—K2—K2^iii^	43.88 (5)
O1—K1—C6	64.38 (6)	O5^vii^—K2—K2^iii^	120.53 (5)
O5—K1—C6	94.38 (6)	O3^viii^—K2—K2^iii^	106.88 (4)
O3—K1—C6	96.35 (6)	O4^viii^—K2—K2^iii^	75.86 (5)
O2^v^—K1—C1^vi^	90.35 (7)	S2—K2—K2^iii^	133.26 (3)
O2^vi^—K1—C1^vi^	21.59 (6)	S1—K2—K2^iii^	121.19 (2)
O1—K1—C1^vi^	100.67 (6)	S2^iii^—K2—K2^iii^	46.809 (17)
O5—K1—C1^vi^	123.68 (6)	K1—K2—K2^iii^	88.808 (18)
O3—K1—C1^vi^	69.09 (6)	O4—K2—K2^viii^	146.64 (6)
C6—K1—C1^vi^	122.90 (6)	O3—K2—K2^viii^	103.43 (5)
O2^v^—K1—S1	109.19 (5)	O5^vii^—K2—K2^viii^	103.43 (4)
O2^vi^—K1—S1	124.25 (4)	O3^viii^—K2—K2^viii^	38.77 (4)
O1—K1—S1	91.12 (4)	O4^viii^—K2—K2^viii^	38.20 (4)
O5—K1—S1	98.34 (4)	S2—K2—K2^viii^	51.744 (13)
O3—K1—S1	79.31 (4)	S1—K2—K2^viii^	106.540 (19)
C6—K1—S1	30.73 (4)	S2^iii^—K2—K2^viii^	78.70 (2)
C1^vi^—K1—S1	135.80 (5)	K1—K2—K2^viii^	87.075 (19)
O2^v^—K1—O1^vi^	74.35 (6)	K2^iii^—K2—K2^viii^	110.46 (3)
O2^vi^—K1—O1^vi^	41.03 (5)	O4—K2—K1^vii^	72.51 (5)
O1—K1—O1^vi^	122.54 (5)	O3—K2—K1^vii^	137.42 (5)
O5—K1—O1^vi^	133.64 (5)	O5^vii^—K2—K1^vii^	37.15 (4)
O3—K1—O1^vi^	50.52 (5)	O3^viii^—K2—K1^vii^	53.25 (4)
C6—K1—O1^vi^	128.70 (6)	O4^viii^—K2—K1^vii^	110.14 (5)
C1^vi^—K1—O1^vi^	21.87 (5)	S2—K2—K1^vii^	125.77 (2)
S1—K1—O1^vi^	127.12 (4)	S1—K2—K1^vii^	129.17 (2)
O2^v^—K1—S2	153.59 (5)	S2^iii^—K2—K1^vii^	60.694 (17)
O2^vi^—K1—S2	74.66 (4)	K1—K2—K1^vii^	177.62 (2)
O1—K1—S2	67.40 (4)	K2^iii^—K2—K1^vii^	93.565 (18)
O5—K1—S2	115.75 (4)	K2^viii^—K2—K1^vii^	91.962 (18)
O3—K1—S2	84.23 (4)	C6—S1—K2	91.16 (9)
C6—K1—S2	29.79 (5)	C6—S1—K1	69.92 (8)
C1^vi^—K1—S2	93.14 (5)	K2—S1—K1	82.14 (2)
S1—K1—S2	52.585 (17)	C6—S2—K2	92.67 (8)
O1^vi^—K1—S2	100.79 (4)	C6—S2—K1	65.51 (8)
O2^v^—K1—K2	106.61 (4)	K2—S2—K1	80.519 (19)
O2^vi^—K1—K2	84.08 (4)	C6—S2—K2^viii^	171.04 (10)
O1—K1—K2	114.65 (4)	K2—S2—K2^viii^	81.447 (18)
O5—K1—K2	146.95 (4)	K1—S2—K2^viii^	119.65 (2)
O3—K1—K2	39.52 (4)		
			
O2—C1—C2—N	160.5 (2)	C4—C3—N—C2	−8.5 (3)
O1—C1—C2—N	−21.9 (3)	O2—C1—O1—K1	−116.4 (2)
K1^i^—C1—C2—N	95.0 (3)	C2—C1—O1—K1	66.3 (3)
O2—C1—C2—C5	−84.9 (3)	K1^i^—C1—O1—K1	−79.32 (18)
O1—C1—C2—C5	92.6 (3)	O2—C1—O1—K1^i^	−37.0 (2)
K1^i^—C1—C2—C5	−150.4 (2)	C2—C1—O1—K1^i^	145.6 (2)
N—C3—C4—C5	28.1 (3)	O1—C1—O2—K1^ii^	−176.93 (17)
C3—C4—C5—C2	−37.1 (3)	C2—C1—O2—K1^ii^	0.5 (3)
N—C2—C5—C4	31.5 (3)	K1^i^—C1—O2—K1^ii^	135.8 (2)
C1—C2—C5—C4	−88.5 (3)	O1—C1—O2—K1^i^	47.3 (3)
S1—C6—N—C2	173.28 (17)	C2—C1—O2—K1^i^	−135.35 (18)
S2—C6—N—C2	−4.1 (3)	N—C6—S1—K2	178.61 (19)
K1—C6—N—C2	87.7 (2)	S2—C6—S1—K2	−4.09 (15)
S1—C6—N—C3	0.5 (3)	K1—C6—S1—K2	−81.19 (4)
S2—C6—N—C3	−176.82 (19)	N—C6—S1—K1	−100.2 (2)
K1—C6—N—C3	−85.1 (2)	S2—C6—S1—K1	77.11 (14)
C1—C2—N—C6	−68.8 (3)	N—C6—S2—K2	−178.53 (19)
C5—C2—N—C6	171.9 (2)	S1—C6—S2—K2	4.15 (15)
C1—C2—N—C3	104.8 (2)	K1—C6—S2—K2	78.32 (4)
C5—C2—N—C3	−14.5 (3)	N—C6—S2—K1	103.2 (2)
C4—C3—N—C6	165.0 (2)	S1—C6—S2—K1	−74.17 (14)

Symmetry codes: (i) *x*−1/2, −*y*+1/2, −*z*+2; (ii) *x*−1, *y*, *z*; (iii) *x*+1/2, −*y*+3/2, −*z*+2; (iv) *x*, *y*−1, *z*; (v) *x*+1, *y*, *z*; (vi) *x*+1/2, −*y*+1/2, −*z*+2; (vii) *x*, *y*+1, *z*; (viii) *x*−1/2, −*y*+3/2, −*z*+2.

### Hydrogen-bond geometry (Å, º)

**Table:**

*D*—H···*A*	*D*—H	H···*A*	*D*···*A*	*D*—H···*A*
O3—H9···O1^vi^	0.83 (2)	1.93 (2)	2.752 (3)	170 (4)
O3—H8···S2^v^	0.81 (2)	2.57 (2)	3.3123 (19)	153 (3)
O4—H11···O2^ix^	0.80 (2)	2.22 (2)	3.000 (3)	166 (4)
O5—H13···O1	0.81 (2)	1.99 (3)	2.724 (3)	150 (3)
O5—H12···S1^x^	0.81 (2)	2.55 (2)	3.341 (2)	163 (3)

Symmetry codes: (v) *x*+1, *y*, *z*; (vi) *x*+1/2, −*y*+1/2, −*z*+2; (ix) *x*+1, *y*+1, *z*; (x) −*x*+1, *y*−1/2, −*z*+3/2.
